# Engineering entangled microwave photon states through multiphoton interactions between two cavity fields and a superconducting qubit

**DOI:** 10.1038/srep23646

**Published:** 2016-04-01

**Authors:** Yan-Jun Zhao, Changqing Wang, Xiaobo Zhu, Yu-xi Liu

**Affiliations:** 1 Institute of Microelectronics, Tsinghua University, Beijing, 100084, China; 2 Institute of Physics, Chinese Academy of Sciences, Beijing, 100190, China; 3 Tsinghua National Laboratory for Information Science and Technology (TNList), Beijing 100084, China

## Abstract

It has been shown that there are not only transverse but also longitudinal couplings between microwave fields and a superconducting qubit with broken inversion symmetry of the potential energy. Using multiphoton processes induced by longitudinal coupling fields and frequency matching conditions, we design a universal algorithm to produce arbitrary superpositions of two-mode photon states of microwave fields in two separated transmission line resonators, which are coupled to a superconducting qubit. Based on our algorithm, we analyze the generation of evenly-populated states and NOON states. Compared to other proposals with only single-photon process, we provide an efficient way to produce entangled microwave photon states when the interactions between superconducting qubits and microwave fields are in the strong and ultrastrong regime.

Superconducting transmission line resonators can be used as quantum data buses, quantum memories, and single microwave photon detectors^1^,^2^. They usually work in the microwave regime and can also be used as quantum nodes in so-called quantum networks^3^,^4^. It is well known that the entanglement is one of the most important resources for quantum information processing^5^, and microwave photons play a critical role in quantum state control for solid state quantum devices. Therefore, engineering arbitrarily entangled microwave photon states is a very fundamental issue for both solid state quantum information processing and quantum optics^6^ on superconducting quantum chips.

Usually, nonclassical photon states of a single-mode cavity field are generated through the interaction between the cavity field and the two-level atom. The methods of generating nonclassical photon states can be classified into two ways. One is to engineer appropriate Hamiltonians in different evolution durations by tuning experimental parameters when the target state is being generated^7^,^8^,^9^,^10^. The other one is to obtain the target state via appropriately designed measurements^11^. The former one is deterministic, while the latter one is probabilistic and usually has a low probability to succeed. If the nonclassical state is generated using natural atomic systems, the latter method is usually more practical since most of parameters are not possible or not easy to be tuned. However, in artificial atomic systems, the former method is more appropriate because system parameters can be artificially controlled. For example, superconducting quantum circuits (SQCs)^12^,^13^,^14^,^15^,^16^,^17^,^18^,^19^ provide us a very convenient way to deterministically engineer nonclassical states of a single-mode microwave field by varying the system parameters^7^,^8^,^9^,^10^.

The method of deterministically generating entangled photon states using atomic systems can be tracked to that of generating entangled phonon states of two vibrational modes^20^, in a trapped ion interacting with laser fields, by using different sideband transitions. However, the number of steps in such a method^20^ exponentially depends on the maximum phonon numbers. A few proposals were put forward to overcome the exponential dependence of the phonon number by introducing auxiliary atomic energy levels^21^,^22^, using phonon number dependent interactions^23^, or employing multiphonon transitions of high phonon numbers^22^,^24^. These methods have successfully reduced the number of steps into quadratic polynomials of the maximum phonon numbers.

The generation of entangled microwave photon states of two modes using superconducting qubit has been studied^25^,^26^,^27^, where a classically driven superconducting qubit with time-dependent frequency is coupled to two microwave fields in two separated cavities. The interaction Hamiltonian between the superconducting qubit and the cavity fields of two modes is described by the Jaynes-Cummings model. Therefore, there is only single photon transition in each step. However, the photon-number-dependent Stark effects^25^,^26^,^27^ induced by the qubit-field coupling make it possible to independently implement operations for photon states. Thus, the number of steps also quadratically depends on the maximum photon number.

It has been shown that the superconducting qubit and the cavity field can have both transverse and longitudinal couplings when the inversion symmetry of the qubit potential energy is broken^28^,^29^. The longitudinal coupling can induce multiphoton transitions^30^ in different sidebands as in trapped ions^31^,^32^ and thus arbitrary photon states of a single-mode cavity field can be more conveniently engineered^30^. Motivated by studies^25^,^26^,^27^,^28^,^29^,^30^, we study a method to generate entangled microwave photon states in two separated cavities coupled by a superconducting qubit using multiphoton transitions. We first show that the longitudinal couplings can induce two-mode multiphoton processes similar to those in trapped ions^33^, and then study an efficient way to generate superposed two-mode photon states.

The paper is organized as below. In Sec. Theoretical Model and Sideband Excitations, an effective Hamiltonian, similar to that of trapped ions with two vibrational modes^33^, is derived, and then different sideband transitions are discussed. In Sec. Algorithm for State Generation, a new algorithm is introduced to generate arbitrary superpositions of two-mode photon states. In Sec. Minimizing the Effect of Unwanted Terms, we discuss how to choose parameters to obtain a high fidelity of the target state. In Sec. Environmental Effect on Target States, we numerically study the effects of both imperfect control pulses and the environment on the generated target state. In Sec. Discussions, the advantages and experimental feasibility of our method are discussed. Finally, we summarize our results in Sec. Conclusions.

## Theoretical Model and Sideband Excitations

### Basic Hamiltonian

**Figure 1:**
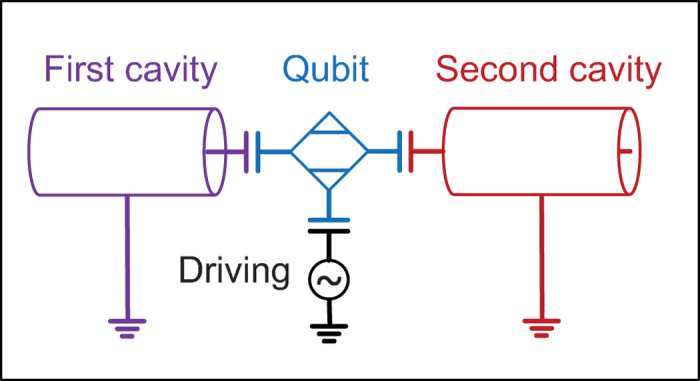
Schematic diagram for a driven qubit (in the middle with the blue color), which is coupled to two single-mode microwave fields of two separated cavities (in the left with the purple color and the right with the red color, respectively). The first cavity field has the frequency *ω*_1_ and the second one has the frequency *ω*_2_. The coupling strength is *g*_1_ (*g*_2_) between the qubit and the first (second) cavity field. The qubit is driven by a classical field (in the middle with the black color) with the frequency 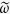 and Rabi frequency Ω.

As schematically shown in Fig. 1, we study a system where a superconducting qubit (SQ), modeled as a two level system, is coupled to two single-mode microwave fields in two separated cavities and driven by a classical field. The system Hamiltonian can be given by


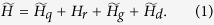


Here, 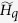 and *H*_*r*_ are the free Hamiltonians of the SQ and the cavity fields, respectively. Moreover, 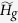 is the interaction Hamiltonian between the SQ and cavity fields, and 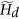 is the interaction Hamiltonian between the SQ and the classical field. In the qubit basis, the qubit Hamiltonian is given by


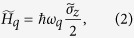


with 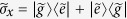 and 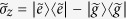. The parameter *ω*_*q*_ is the qubit frequency. The kets 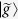 and 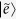 denote the ground and excited states of the qubit, respectively.

The free Hamiltonian of two cavity fields is given by


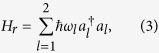


where *a*_*l*_ 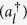 is the annihilation (creation) operator of the *l*th cavity field with its frequency *ω*_*l*_ and *ω*_1_ ≠ *ω*_2_. The interaction Hamiltonian between the qubit and two cavity fields is


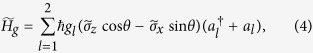


where *g*_*l*_ is the coupling strength between the *l*th cavity field and the qubit, and *θ* is a parameter which depends on the inversion symmetry of the qubit potential energy.

Similarly, the interaction Hamiltonian between the qubit and classical field is given by


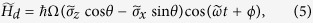


where Ω is the coupling strength (or Rabi frequency) between the qubit and the driving field. The parameters 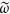 and *ϕ* are the driving frequency and driving phase, respectively.

In Eqs. (4) and (5), when the qubit potential energy possesses inversion symmetry, i.e., cos *θ* = 0, there are only transverse couplings between the qubit and cavity fields^29^. If the rotating wave approximation is further made and there is no driving (Ω = 0), Eq. (1) is reduced to extensively studied Jaynes-Cummings model^6^. When the qubit potential energy possesses a broken inversion symmetry^28^,^29^, i.e., cos *θ* ≠ 0, there are both transverse and longitudinal couplings between the qubit and microwave fields. The broken inversion symmetry of the qubit potential energy can be achieved when the bias charge for the charge qubit or the bias flux for the flux qubit is tuned off the optimal point^28^,^29^. But for the phase qubit, the inversion symmetry of the potential energy is always broken^34^,^35^. Here, we will study a general method and not specify a particular qubit.

We now change the qubit basis into the current basis of the flux qubit or the charge basis of the charge qubit. This is equivalent to diagonalizing the operator 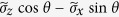. In the new basis, the Hamiltonian in Eq. (1) becomes


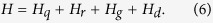


Here, the Hamiltonians *H*_*q*_, *H*_*g*_, and *H*_*d*_ are given by


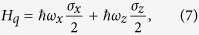



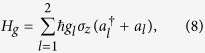



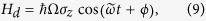


with 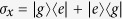, and 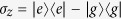. Hereafter, the parameters *ω*_*x*_ = *ω*_*q*_ sin *θ* and *ω*_*z*_ = *ω*_*q*_ cos *θ* are called transverse and longitudinal frequencies of the qubit, respectively. The kets 
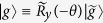
 and 
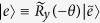
 are persistent current states of the flux qubit or charge states of the charge qubit. Here, 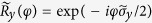 is the rotation operator along the *y*-axis, with 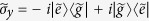. The parameter *ω*_*z*_ ≠ 0 results in longitudinal couplings between the qubit and microwave fields in Eq. (1). Below, we will show that *ω*_*z*_ ≠ 0 can induce two-mode multiphoton processes in the qubit, and then use these multiphton processes to generate arbitrary superpositions of two-mode photon states.

### Multiphoton processes and sideband excitations

To see how the multiphoton processes can be induced by the longitudinal coupling when *ω*_*z*_ ≠ 0, we now apply a unitary transformation


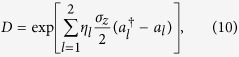


to the Hamiltonian in Eq. (6). Then, we obtain an effective Hamiltonian


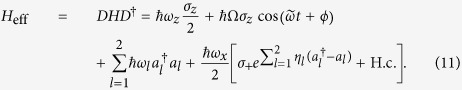


It is clear that *D* is the displacement operator^6^ of two-mode cavity fields. The displacement quantity is *η*_*l*_*σ*_*z*_/2 for the *l*th cavity field. Hereafter, we will call the picture after the operator *D* as the displacement picture. The ratios *η*_*l*_ = 2*g*_*l*_/*ω*_*l*_ are called the Lamb-Dicke parameters in analogy to trapped ions^31^,^32^.

To understand the classical-field-assisted multiphoton transitions of two cavity fields in the qubit, we apply to Eq. (11) a time-dependent unitary transformation


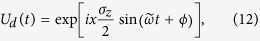


with 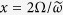. Then, another effective Hamiltonian


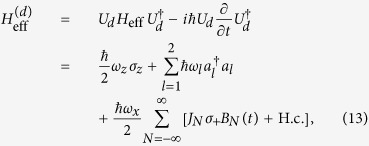


can be derived, with the time-dependent term


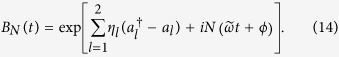


Here, *J*_*N*_ ≡ *J*_*N*_(*x*) is the Bessel function of the first kind. Equation (13) shows that multiphoton transitions with different modes can be controlled by the classical field as in trapped ions^33^.

In the interaction picture with the free Hamiltonian 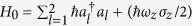, Equation (13) becomes


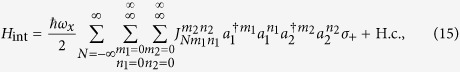


where 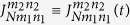 is the coupling strength between the qubit and cavity field with each different transition process, and its algebraic form is


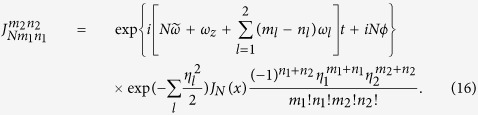


Equation (15) describes the classical-field-assisted two-mode multiphoton processes as in trapped ions^33^. The magnitude of 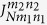 depends on *ω*_*x*_, *x*, and *η*_*l*_. We find


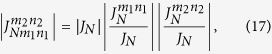


where the properties of 
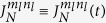
 have been studied in ref. 30. The specific expression of 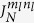 is given by


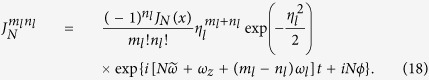


Similarly to Eq. (17), the magnitude of 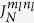 can be rewritten as


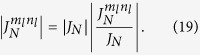


It is clear that 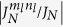 is independent of the reduced driving strength *x*. From Eqs. (17)–(19), 97, 102we know that both 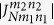 and 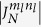 can be changed by adjusting *x* and *η*_*l*_ in a similar way. By introducing new variables *k*_*l*_ = *m*_*l*_ − *n*_*l*_, we expand Eq. (15) in the Fock state basis, and then have


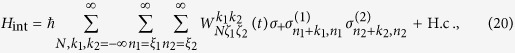


with *ξ*_*l*_ = max{0, −*k*_*l*_} and *ζ*_*l*_ = min{*n*_*l*_, *n*_*l*_ + *k*_*l*_}. Here, 
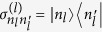
 denotes the ladder operator of the *l*th cavity field. The time-dependent transition element 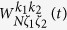 is given by


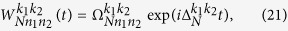


with *n*_1_, *n*_2_ replaced by *ζ*_1_, *ζ*_2_ respectively. The complex transition amplitude 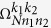 and detuning 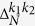 are respectively


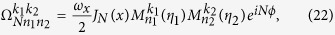



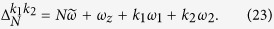


The parameter 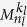 is given by


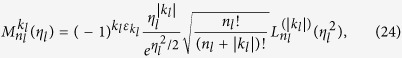


with


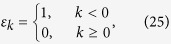



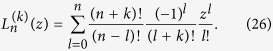


Here, 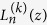 is the generalized Laguerre polynomials. It is clear that the classical-field-assisted multiphoton transitions can be derived from Eq. (20) using different frequency-matching conditions.

### Time evolution operators

We now give detailed discussions on how to engineer two-mode multiphoton processes by tuning the driving field. Let us assume that the driving field is tuned to satisfy the resonant condition


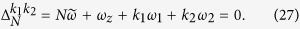


Then Eq. (20) can be reduced to an effective Hamiltonian 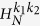 when unwanted terms are neglected, that is,


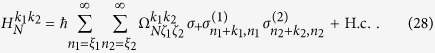


The time evolution operator governed by the Hamiltonian in Eq. (28) is given by


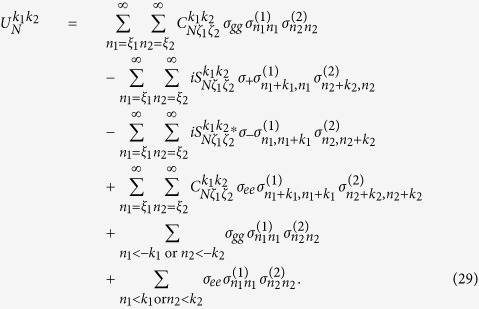


Recall that *ξ*_*l*_ = max{0, −*k*_*l*_} and *ζ*_*l*_ = min{*n*_*l*_, *n*_*l*_ + *k*_*l*_} as defined previously. Here the new parameters used in Eq. (29) are respectively


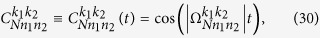



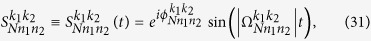



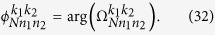


As shown in Eqs. (28) and (29), |*k*_*l*_| photons in the *l*th resonator can be either created if *k*_*l*_ ≥ 0 or annihilated if *k*_*l*_ < 0 while the qubit is flipped up. Similarly, |*k*_*l*_| photons in the *l*th resonator can be either created if *k*_*l*_ < 0 or annihilated if *k*_*l*_ ≥ 0 while the qubit is flipped down. Thus different sideband excitations can be constructed, depending on the values of *k*_1_ and *k*_2_.

Because the Hamiltonian derived in Eq. (15) is similar to that of the trapped ions^24^, the algorithm using two-mode multiphonon processes in trapped ions can be directly applied into our model, and different superpositions of two-mode photons can be generated. As a special case, two-mode Fock states of high photon numbers can in principle be more efficiently generated with just two steps as single-mode Fock states of high photon numbers^30^. However, we here design a new algorithm via different sideband transitions of low photon numbers by tuning the driving field with properly selecting the parameters *ω*_*z*_, *ω*_*x*_, *ω*_*l*_, and *η*_*l*_. The detailed discussions of parameter selection will be given in Sec. 3.

## Algorithm for State Generation

Let us first study a universal algorithm for generating arbitrary two-mode microwave photon states using sideband transitions with the following four Hamiltonians 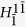, 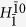, 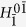, and 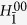. Here, for the compact of notations, we have used 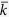 to represent −*k* with *k* > 0. For instance, 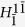 is actually 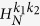 with *N* = −1, *k*_1_ = 1, and *k*_2_ = −1. For different *N*, *k*_1_, and *k*_2_, the interaction Hamiltonian 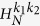 and its time evolution operator 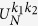 have already been given in Sec. 1. Below, we will first study how to generate the target state by choosing pulse durations, frequencies, and phases of the driving fields at each generation step with different sideband excitations, and then we will apply our algorithm to the generation of NOON states and discuss particular properties of the algorithm.

### Universal algorithm for generating arbitrary two-mode microwave photon states

We note that the state generation in our algorithm is studied in the displacement picture with the unitary transformation as shown in Eq. (10). The arbitrary quantum states, we expect to be generated, is written as


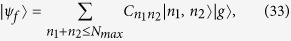


where 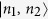 means that the first and second cavities contain *n*_*l*_ and *n*_2_ photons, respectively, and 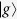 means that the qubit is in the ground state. Besides, *N*_max_ and 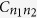 mean the maximum photon number and the probability amplitude on the state 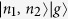, respectively. We assume that the system is initially in the state


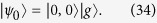


We suppose the target state 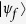 can be generated by alternately switching on and off the two-mode transitions 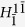, 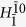, 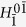, and 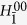. With the designed time evolution operators, the state generation procedure can be represented by,


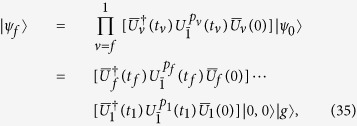


where 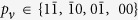 denotes the transition type for the *ν*th step, and *t*_*ν*_ is the time duration for the *ν*th step. The time evolution operator 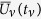 is given by


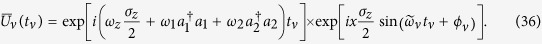


As discussed above, the transitions of different types can be achieved by changing the frequency 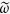 of the driving field, which is denoted by 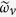 for the *ν*th step. The phase of the driving field for the *ν*th step is denoted by *ϕ*_*ν*_. We can express Eq. (35) in another equivalent form of iteration,


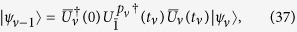


with |*ψ*_0_〉 and |*ψ*_*f*_〉 given in Eqs. (34) and (33), respectively. The ket |*ψ*_*ν*_〉 is the state after the *ν*th step. We note that the subscript *f* of |*ψ*_*f*_〉 in Eq. (35) denotes the number of the final step. Equation (37) means that the initial state is restored from the target state by a composition of sideband transitions with proper time durations, frequencies and phases of driving fields. It is a recursion algorithm.

Without loss of generality, we use the maximum photon number *N*_max_ = 2 as an example to show our algorithm. The more general case with arbitrary *N*_max_ is given in the supplementary material. The detailed steps for generating the target state


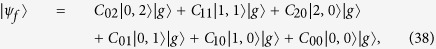


with *N*_max_ = 2 using our recursion algorithm are described as the following four procedures.

**Figure 2:**
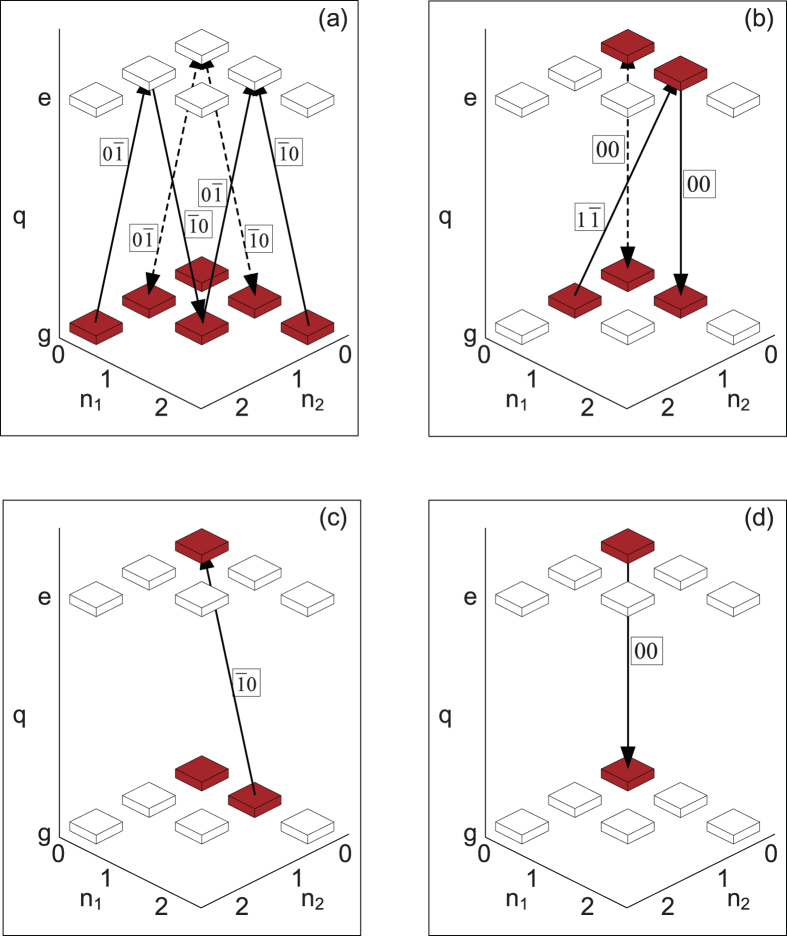
Universal algorithm for generating arbitrary two-mode superposition state with the maximal photon number *N*_max_ = 2. The *n*_1_ and *n*_2_-axis respectively denote the photon number of the first and second mode. The two-mode photon state is denoted by |*n*_1_, *n*_2_〉. The qubit state is represented by the *q*-axis with *q* = *g* or *e* respectively denoting the ground state |*g*〉 or excited state |*e*〉. The state component |*n*_1_, *n*_2_〉|*q*〉 is represented by a block at the location (*n*_1_, *n*_2_, *q*). If a state component is occupied, we color the corresponding block with red; otherwise, the block is left uncolored. The arrows respectively represent the “
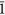
0”, “0
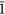
”, “1
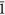
”, and “00” transitions with transition types labeled aside them. The solid arrow indicates a desired population transfer from the starting state to the end state, while the dashed arrow indicates the inevitable oscillation when the desired population transfer is implemented. The inevitable oscillations have no effect on the results or fidelity of the target state. (**a**) Schematic diagram for transferring the populations on states |0, 2〉|*g*〉, |1, 1〉|*g*〉, and |2, 0〉|*g*〉 to the state |1, 0〉|*e*〉. This is achieved by consecutively using “0
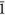
”, “
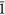
0”, “0
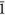
”, and “
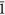
0” transitions. (**b**) Schematic diagram for transferring populations on states |0, 1〉|*g*〉 and |1, 0〉|*e*〉 to the state |1, 0〉|*g*〉. This is achieved by consecutively using “1
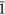
” and “00” transitions. (**c**) Schematic diagram for transferring the population on the state |1, 0〉|*g*〉 to the state |0, 0〉|*e*〉. This is achieved by using a “
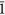
0” transition. (**d**) Schematic diagram for transferring the population on the state |0, 0〉|*e*〉 to the state |0, 0〉|*g*〉. This is achieved by using a “00” transition.

**Procedure (i)**. As schematically shown in Fig. 2(a), from the final state |*ψ*_*f*_〉, we first transfer the populations in the state space spanned by {|*n*_1_, *n*_2_〉|*g*〉|*n*_1_ + *n*_2_ = 2} to the state |1, 0〉|*e*〉. This procedure consists of four steps as schematically shown in below


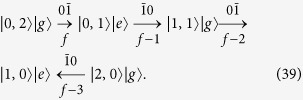


In Eq. (39), the transition type and step number is labeled respectively above and below the arrow. The arrow points to the direction of the population transfer. In Step *ν*, the population transfer is accomplished by properly tuning 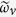, *t*_*ν*_, and *ϕ*_*ν*_. After this procedure, we obtain the state |*ψ*_*f*−4_〉 which only has popupaltions in the space 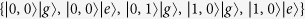. We note that two additional oscillations


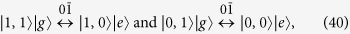


will also occur inevitably when the population transfer from the state 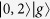 to the state 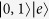 is implemented. But they do not cause population leakage outside the original space and no extra steps should be taken for them. Thus these oscillations have no effect on the results or fidelity of the target state. For this procedure, these oscillations are schematically shown by dashed arrows in Fig. 2(a). Such oscillations can also occur in the following procedures and are shown by dashed arrows.

**Procedure (ii)**. As schematically shown in Fig. 2(b), starting from the state |*ψ*_*f*−4_〉, we need to transfer the populations in the state space spanned by {|0, 1〉|*g*〉, |1, 0〉|*e*〉} to the state |1, 0〉|*g*〉. This procedure consists of following two steps


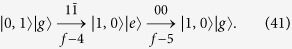


After this procedure, we obtain the state |*ψ*_*f*−6_〉, which only has popupaltions in the space 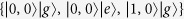.

**Procedure (iii)**. This procedure is similar to Procedure (i). As schematically shown in Fig. 2(c), starting from the state |*ψ*_*f*−6_〉, here we need to transfer the population on the state |1, 0〉|*g*〉 to the state |0, 0〉|*e*〉. This procedure consists of only one step as below


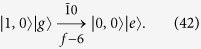


After this procedure, we obtain the state |*ψ*_*f*−7_〉, which only has popupaltions in the space 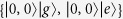.

**Procedure (iv)**. This procedure is similar to the Procedure (ii). As schematically shown in Fig. 2(d), starting from the state |*ψ*_*f*−7_〉, we need to transfer the population on the state |0, 0〉|*e*〉 to the state |0, 0〉|*g*〉. This procedure only consists of one step as below


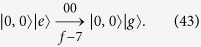


We thus obtain the state |*ψ*_*f*−8_〉 = |0, 0〉|*g*〉.

Therefore, the target 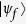 can be generated from the initial state 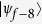 using inverse processes from the Procedure (iv) to the Procedure (i). We note 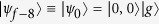. Thus, we obtain the total step number *f* = 8 by setting *f* − 8 = 0. Therefore, the generation of the target state with *N*_max_ = 2 needs 8 steps.

Our algorithm takes a quadratic number of steps while an exponential one is required in ref. 20. Let us now analyze the reason. Our algorithm employs four interaction Hamiltonians, 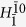, 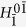, 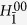, and 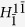 given in Eq. (28). However, four interaction Hamiltonians “
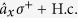
”, “
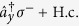
”, “*σ*^−^ + *σ*^+^”, and “
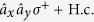
” are employed in ref. 20. The former three interaction Hamiltonians between our algorithm and those in ref. 20 are qualitatively identical since they convert the same number of bosons for either mode when the two-level system is excited. However, the last ones show fundamental difference between our algorithm and that in ref. 20, because ours creates one boson (photon) of one mode but annihilate one boson (photon) of the other when the two-level system is excited. But in ref. 20, one boson for both modes can be simultaneously created when the two-level system is excited. This difference is critical for us to design an algorithm which can keep track of the populations with a constant total boson (photon) number. Therefore, there is no population leakage outside the original space. However, the algorithm in ref. 20 has population leakage. Obviously, if the last interaction in ref. 20 is changed to “
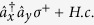
”, a theoretically equivalent algorithm to ours can also be developed. In this sense, our algorithm can be regarded as the improved version of that in ref. 20.

### Calculation of controllable parameters

Let us now study how to choose the pulse duration *t*_*ν*_, the frequency 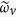 and phase *ϕ*_*ν*_ of the driving field to generate a target state in the *ν*th step for different types of transitions.

We suppose that the population transfer is taken as following


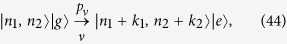


in the *ν*th step, where the transition type *p*_*ν*_ = *k*_1_*k*_2_ should be switched on based on the previous discussions. Thus the driving frequency is taken as


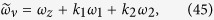


from the resonant condition in Eq. (27). By introducing the notations


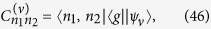



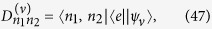


then from Eq. (37), we need to solve the equation,


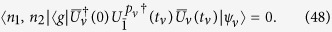


We thus have the explicit solution for the pulse duration *t*_*ν*_ as


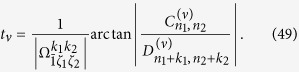


The phase of the driving field is determined by


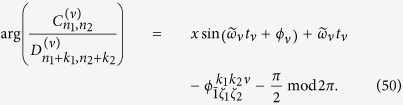


Here, the notation 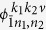 is the value of 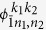 for the *ν*th step, which is given in Eq. (32) and depends on *ϕ*_*ν*_. Still recall *ζ*_*l*_ = min{*n*_*l*_, *n*_*l*_ + *k*_*l*_} with *l* = 1, 2.

Similarly, if the population transfer is taken as


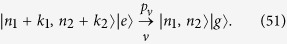


in the *ν*th step. The explicit solution for *t*_*ν*_ is then


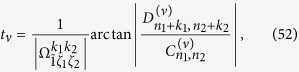


and the phase of the driving field is determined by


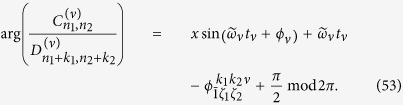


According to the target state, the time duration, frequency and phase of the driving field for each step can be calculated using above equations. For example, if the “00” transition is used in the 3rd step, then we use Eq. (52) and Eq. (53) to obtain *t*_3_ and *ϕ*_3_ by setting *ν* = 3.

### Application to NOON states

As an example, we now apply our algorithm to the generation of the NOON state, i.e., the target state is


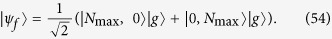


**Figure 3:**
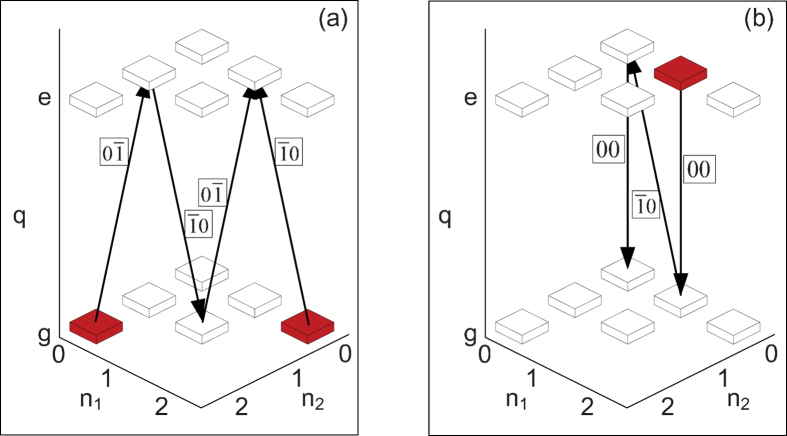
Application of the general algorithm to generating the NOON state. The notations are the same as those in Fig. 2. (**a**) Schematic diagram for transferring the population in the space {|0, 2〉|*g*〉, |2, 0〉|*g*〉} to the state |1, 0〉|*e*〉. This is achieved by consecutively using “0
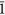
”, “
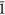
0”, “0
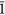
”, and “
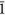
0” transitions. (**b**) Schematic diagram for transferring the population on the state |1, 0〉|*e*〉 to the state |0, 0〉|*g*〉. This is achieved by consecutively using “00”, “
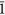
0”, and “00” transitions.

The recursion algorithm restoring 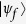 to the vacuum state |0, 0〉|*g*〉 is schematically shown in Fig. 3 for the maximum photon number *N*_max_ = 2. In Fig. 3(a), we can find that all the populations in the Hilbert space spanned by {|0, 2〉|*g*〉, |2, 0〉|*g*〉} can be transferred to the state |1, 0〉|*e*〉 by consecutively using transitions “0
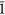
”, “
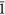
0”, “0
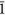
”, and “
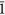
0”, i.e.,


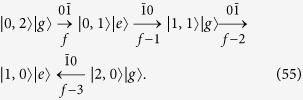


After this procedure, as schematically shown in Fig. 3(b), all the populations on the state |1, 0〉|*e*〉 can be transferred to the state |0, 0〉|*g*〉 by consecutively using transitions “00”, “
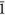
0”, and “00”, i.e.,


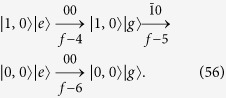


The total step number is thus *f* = 7 for generating the NOON state 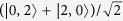.

More generally, given an arbitrary *N*_max_, the total step number for generating the NOON state in Eq. (54) is


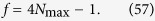


The step number for generating NOON state has been greatly reduced in comparison with that for generating an arbitrary state (see Equation (16) in the supplementary information). Obviously, the NOON state can be generated without using the “1
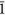
” transition. If we assume the Lamb-Dicke parameter 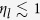, which is usually the case even in the ultrastrong regime in superconducting circuit QED systems^36^,^37^,^38^. From Eq. (22), we have the Rabi frequencies 
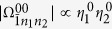
, 
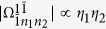
, 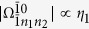, and 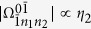. Thus, the transition “1
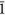
” generally takes more time among the four types of transitions employed by us. Therefore, our algorithm may show a better efficiency for generating NOON sates than generating arbitrary entangled states. This is especially true when the maximal photon number *N*_max_ is higher and the Lamb-Dicke parameter *η*_*l*_ is smaller.

## Minimizing the Effect of Unwanted Terms

### Theoretical analysis

In all of the above studies, we make an approximation that all unwanted terms have been neglected. However, these neglected terms will affect the fidelity of the prepared target state. Let us now discuss how to minimize the effect of these unwanted terms in Eq. (20) on the target state by choosing appropriate parameters. In principle, the effects of these unwanted terms can be perfectly removed by pulse calibration techniques. Here, we study a method to minimize the effect of these unwanted terms by choosing the parameters when the pulse calibration cannot be used.

In our algorithm, we have used four interactions 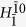, 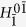, 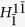, and 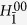, all of them are constructed by the terms with the Bessel function 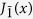 in Eq. (20). Here, in the subscript of the Bessel function, we also use 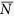 to denote −*N* if *N* > 0. We hope to suppress all the terms with the Bessel functions *J*_*N*′_(*x*) for *N*′ ≠ −1. We focus on the case 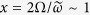 considering possible experimental conditions. In this case, only lower order Bessel functions *J*_0_(*x*), *J*_±1_(*x*), and *J*_±2_(*x*) play significant roles. Thus, we need only to find proper parameters such that the effect of the terms with *J*_0_(*x*), *J*_1_(*x*), and *J*_±2_(*x*) are negligibly small. Our idea is to make those terms nonresonant by properly choosing the parameters *ω*_*x*_ and *ω*_*z*_ of the qubit, and frequencies *ω*_1_ and *ω*_2_ of two microwave modes. That is, we assume that the frequency of the *l*th cavity mode satisfies


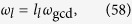


where *l*_*l*_ is a positive integer and *ω*_gcd_ is the greatest common divisor of *ω*_1_ and *ω*_2_. Assuming that the “*k*_1_*k*_2_” transition is switched on, i.e., the transition detuning 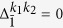, then from Eq. (23), the frequency 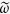 of the driving field must satisfy the condition


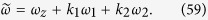


From Eqs. (23), (58), and (59), the detuning of the term with *N*′, 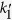, 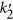 is then given by


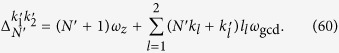


Thus the terms with the Bessel function *J*_1_(*x*) will have the detuning


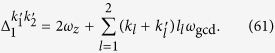


We expect that the terms with *J*_1_(*x*) are nonresonant. Thus, the relation that 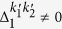 must hold. A simple but sufficient condition is


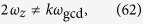


where *k* is an integer. Similarly, for the terms with *J*_0_(*x*), *J*_2_(*x*), and 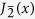, the sufficient conditions can be given by


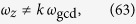



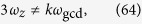



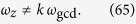


The conditions in Eqs. (62)–(65), 165, 166, 167 can be summarized as


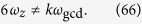


We can also assume that the longitudinal frequency *ω*_*z*_ of the qubit is


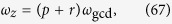


where *p* is the integer part, and *r* is the fraction part. To meet Eq. (66), there should be


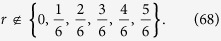


The nonresonant terms with *J*_1_, *J*_0_, and *J*_±2_ still have effect on the desired time evolution. These effects can be further eliminated by decreasing the stark shifts caused by the terms with 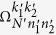 in Eq. (22). The ideal case is


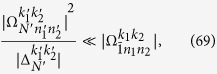


or equivalently,


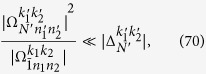


for *N*′ = 0, 1, ±2, 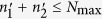, and *n*_1_ + *n*_2_ ≤ *N*_max_, where the constraint condition for *n*_*l*_ and 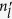 denotes the working space of our algorithm. Equation (69) means that the stark shifts should be negligibly smaller than the Rabi frequencies for state generation. Considering that *N*_max_ is the maximum photon number of the target state, and using Eq. (60) and Eq. (67), we can obtain


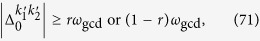



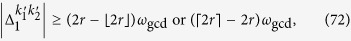



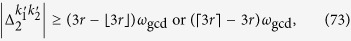



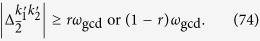


Here, 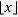 means *x* rounded down and 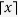 means *x* rounded up. We thus reduce Eq. (70) to


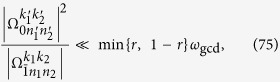



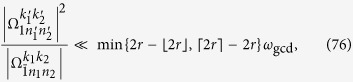



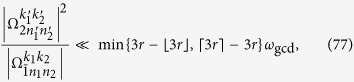



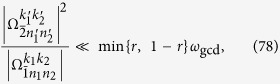


a condition much stronger than Eq. (70). If Eqs. (75)–(78), 183, 184, 185 are fulfilled, the nonresonant terms can in principle be suppressed. We know from Eq. (22) that Eqs. (75)–(78), 183, 184, 185 can be satisfied if, for example, the parameter *ω*_*x*_ of the qubit is tuned sufficiently small, assuming that the reduced driving frequency *x* and Lamb-Dicke parameters *η*_*l*_ have been appropriately chosen.

Beside the terms with Bessel functions 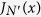 where *N*′ ≠ −1, there are also unwanted terms with the Bessel function 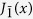, which, however, also satisfy the resonant condition


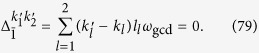


Here, we have used Eqs. (59) and (60) to obtain Eq. (79). The Lamb-Dicke parameters satisfy the condition 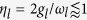 for circuit QED systems even in the ultrastrong regime^36^,^37^,^38^. From Eq. (58), we know that *l*_1_ and *l*_2_ are coprime numbers. We can further make *l*_1_ (or *l*_2_) sufficiently large. Thus the unwanted resonant terms will possess large 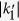 (or 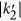). In this way, the effects of these terms will be suppressed due to the exponential decrease via the term 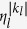 in Eq. (22). The condition, that the term 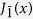 is negligibly small, can be summarized as that *l*_1_ and *l*_2_ should satisfy


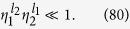


We now summarize the condition that minimizes the effects of unwanted terms. The parameter *ω*_*z*_ of the qubit should satisfy Eq. (67) and Eq. (68). However, the parameter *ω*_*x*_ of the qubit is mainly constrained by current experiments. For example, typical values of *ω*_*x*_/2*π* are in the range 1 ~ 5 GHz. The frequencies of the cavity modes *ω*_*l*_ should satisfy Eq. (58) and Eq. (80). The values of the reduced driving frequency 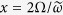 and Lamb-Dick parameter *η*_*l*_ = 2*g*_*l*_/*ω*_*l*_ should satisfy Eq. (70) or stronger conditions Eqs. (75)–(78), 183, 184, 185. Appropriate values of *x* and *η*_*l*_ can be obtained via numerical simulations, which will be discussed below in Sec. 3.

### Numerical simulations

We now further numerically simulate the effect of the unwanted terms on the generation of target states by using examples of generating the following two target states


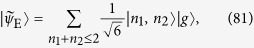



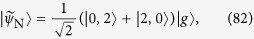


for some given parameters. It is obvious that 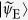 is an entangled state where every state component is evenly occupied. We thus call 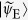 the evenly-populated state. The state 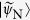 is a two-photon NOON state^5^. Both 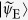 and 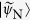 possess a maximum photon number *N*_max_ = 2. The fidelities for generating these two states 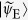 and 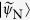 are defined as


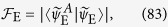



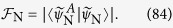


Here, 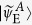 and 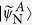 are respectively the actually generated states via the total Hamiltonian in Eq. (6).

We now determine the detailed experimental parameters. From Eq. (67) and Eq. (68), we set *r* = 3/4, *p* = 9, and *ω*_gcd_/2*π* = 2 GHz, which corresponds to *ω*_*z*_/2 = 19.5 GHz. From Eq. (70) or Eqs. (75)–(78), the parameter *ω*_*x*_/2*π* should be made smaller, e.g., we set *ω*_*x*_/2*π* = 1.2 GHz. The frequency of the *l*th cavity, i.e., *ω*_*l*_, is determined by Eq. (58) and (80). Since the microwave fields are usually of several gigahertz, here we set *l*_1_ = 3, and *l*_2_ = 4, thus yielding *ω*_1_/2*π* = *l*_1_*ω*_gcd_/2*π* = 6 GHz and *ω*_2_/2*π* = *l*_2_*ω*_gcd_/2*π* = 8 GHz. The Lamb-Dicke parameters for the first and second cavity modes are set to be identical, i.e.,


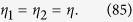


We vary the Lamb-Dick parameter *η* and the reduced driving frequency 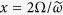 to simulate the effect of the unwanted terms on the fidelity of the expected target states in Eqs. (81) and (82). The pulses are taken according to the calculation of Sec. Calculation of controllable parameters. That is, in the *ν*th step, we use a sinusoidal driving with the driving frequency 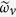. Since the sinusoidal driving lasts for a duration *t*_*ν*_, the driving field can be considered as square-windowed sinusoidal signal and thus, strictly speaking, is not delta-shaped in the spectrum. The simulation results for generating target states in Eq. (81) and Eq. (82) are listed in Tables 1 and 2, respectively. We can easily find that larger reduced driving strengths *x* and Lamb-Dick parameters *η* can usually make the fidelity higher. For the evenly-populated state 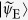, the largest fidelity 0.939 can be obtained at *x* = 1.7571 and *η* = 0.3714. However, for the NOON state 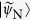, the largest fidelity 0.92 can be obtained at *x* = 2 and *η* = 0.5429.

## Environmental Effect on Target States

In the above, we only discuss the effect of unwanted terms on the generation of target states. We now study the effect of dissipation on the fidelities of target states by numerical simulation for given parameters. When the environmental effect is included, the dynamical evolution of the SQC can be described by the master equation


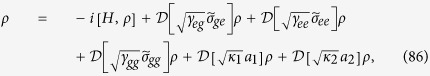


where *ρ* and *H* are the reduced density operator and the Hamiltonian of the whole system, respectively. The total Hamiltonian has been given in Eq. (6). The compact notation 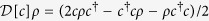 represents the Lindblad-type dissipation. We have noted that {|*g*〉,|*e*〉} is the basis of *σ*_*z*_, but the qubit dissipation is determined by the qubit basis 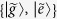. The ground (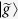) and excited (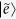) states of the qubit are given by the eigenstates of Eq. (2). If we define


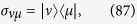


with *ν* = *g*, *e* and *μ* = *g*, *e*, and also define


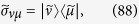


with 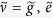 and 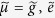. We can easily verify


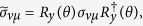


where *R*_*y*_(*θ*) = exp(−*iθσ*_*y*_/2), and *θ* = arc tan(*ω*_*x*_/*ω*_*z*_). In Eq. (86), *γ*_*eg*_ is the pure-relaxation rate from the qubit excited state to the ground state. Besides, *γ*_*gg*_ and *γ*_*ee*_ are the pure-dephasing rates originating from disturbed qubit eigenstates. The decay rates of the first and the second cavity fields are denoted by *κ*_1_ and *κ*_2_, respectively.

Using parameters in Sec. 3 and taking the reduced driving strength 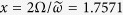 and Lamb-Dicke parameter *η* = 0.3714 from Tables 1 and 2, we find that the highest fidelity 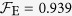 is achieved for generating the evenly-populated state 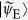 in Eq. (81), and a high fidelity 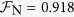 is also reached for generating the NOON state 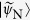 in Eq. (82).

We now assume that the decay rates in Eq. (86) are taken as *γ*_*gg*_/2*π* = 0, *γ*_*ee*_/2*π* = 2 MHz, and *γ*_*eg*_/2*π* = *κ*_1_/2*π* = *κ*_2_/2*π* = 1 MHz. We assume that the density operators 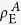 and 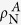 are the actually generated states for the target states 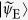 and 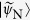. Then the fidelities can be redefined as


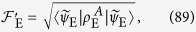



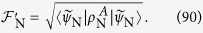


We perform numerical simulations using the above parameters and obtain 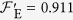 and 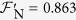. The total time for generating 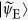 is *T*_E_ = 8.9561 ns and that for generating 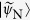 is *T*_N_ = 10.4451 ns. Both *T*_*E*_ and *T*_N_ are too small to induce significant decoherence at the decay rates specified by us. Thus, the fidelity losses induced by dissipation are fairly small, which are 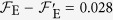 for the evenly-populated state 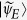 and 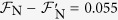 for the NOON state 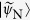. In ref. 39, the experimentally demonstrated 2-photon NOON state is of a fidelity between 0.69 and 0.72 by our definition of fidelity. Thus, it is lower than our result 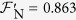.

## Discussions

We now discuss the advantages and disadvantages between our methods and the previous ones^20^,^21^,^22^,^23^,^24^,^25^,^26^,^27^ for generating arbitrarily entangled states of two microwave fields or two vibrational modes.

The brief comparison between these methods is listed in Table 3. In detail, ref. 20 provided an algorithm to generate arbitrarily entangled states of two vibrational modes. But due to population leakage outside the original space, it takes an exponential complexity of the number of steps. The succeeding proposals^21^,^22^,^23^,^24^,^25^,^26^,^27^ overcome the exponential drawback in several ways: (1) A third atomic level is used to shield oscillations that cause population leakage^21^,^22^. But the disadvantage is that higher energy levels of systems usually have larger decay rates, which inevitably reduce the fidelities of the target states. (2) Boson-number-dependent Stark effects are used to realize independent operations of particular states^23^,^25^,^26^,^27^. But the disadvantage is that the detunings of nonresonant terms are usually less by one order of the coupling strengths between the two-level system and boson modes. This means that the Rabi frequencies are smaller, and the longer generation time is required. (3) Multiphoton processes of high photon number are used to shield oscillations that cause population leakage or reduce the number of steps^22^,^24^. But the disadvantage is that if the coupling strengths between the atom and cavity fields are not high enough, then the Rabi frequencies become small, especially for states with high photon numbers, which obviously indicates longer generation time.

Besides the advantage that there is no population leakage, our method has also the following advantages compared with previous ones^21^,^22^,^23^,^24^,^25^,^26^,^27^: (1) It only uses the two energy levels of the qubit. Thus, the fidelities of the target states should be higher because there is no other auxiliary energy levels. (2) The detunings of the nonresonant terms are in the order of the resonator frequencies. They are usually bigger than the coupling strengths between the qubit and resonator modes. Thus the Rabi frequency can be made bigger than those using boson-number-dependent Stark effects. (3) We use multiphoton processes of low photon number, i.e., one photon at most is converted for either mode. Thus the Rabi frequency can be bigger than those using multiphoton processes of higher photon number, especially when the coupling strengths between the qubit and cavity modes are not very big. Of course, stronger couplings will further enhance the Rabi frequencies and hence reduce the generation time.

We point out that the real supercoducting qubit circuits are mutilevel systems, the information leakage to higher levels is not avoidable. However, the leakage can be neglected when the transition frequency between the first excited state and the second excited state is much larger than the qubit frequency. For example, in the flux qubit circuits, due to its large anharmonicity of energy levels, the information leakage is negligibly small. However, for the transmon and phase qubit, the anharmonicity is very weak. Thus, the pulse should be carefully calibrated to avoid information leakage to higher levels. The pulse calibration can be done as in ref. 40.

We now compare the differences between our algorithm and other ones for generating NOON states. Ref. 41 uses mutliphoton processes to generate NOON states. In superconducting systems, this means a low generation efficiency if the Lamb-Dicke parameter is not sufficiently big. Ref. 42 uses synchronization technology to generate NOON states, but the time duration for synchronization between two steps can be quite long and there exists inevitably information leakage. Ref. 43 and its experimental realization^39^ use two phase qubits with three active energy levels to generate NOON states of two cavity modes. The experimental setup is complex and the high energy levels of qubits will reduce the decoherence time. Ref. 25 uses photon number-dependent Stark effects to achieve independent operations. Thus the Rabi frequency is smaller than the qubit-cavity coupling strengths. Ref. 44 requires that two qubits be initially prepared in a Bell state and finally get decoupled from the qubits and cavity fields. Ref. 45 uses one qubit but still needs one additional level to shield unwanted resonances. More recently, ref. 46 uses one qubit of four levels which resonantly interacts with two resonators simultaneously to speed up the generation process of NOON states.

When applied to generating NOON states, our algorithm has new features besides the common advantages for generating arbitrary two-mode photon states: (1) Only carrier processes^31^ and one-photon processes are used. In this case, even though the coupling strengths between the qubit and cavity modes are small, large Rabi frequencies can still be obtained. (2) The number of steps is reduced to linear dependence on the maximum photon number. These advantages indicate less generation time and thus guarantee a higher efficiency than preceding methods.

Now we discuss the experimental feasibility of our scheme. Tables 1 and 2 show that without pulse calibration, higher fidelities can be achieved at bigger Lamb-Dicke parameters *η* and reduced driving frequencies *x*. These values are already in the ultrastrong regime. Ref. 36 has reported ultrastrong couplings between three resonator modes and a flux qubit, where the Lamb-Dicke parameter *η* can reach as high as 0.236. In the ultrastrong regime, Rabi frequencies can be made to approach the magnitude of *ω*_*x*_, which usually ranges from 1 to 5 GHz. The decay rates of the qubit and cavity fields are usually in the magnitude of megahertz. Thus the dissipation has small effect on the fidelities of target states. For singe-mode microwave fields, Fock states with up to six photons^9^ and Fock state superpositions^10^ have been experimentally demonstrated using phase qubits. The NOON state up to 3 photons has also been experimentally reported^39^. We thus hope that our proposal is also experimentally feasible in the near future.

## Conclusions

In summary, we have proposed an approach to generate arbitrary superpositions of photon states of two microwave fields in two separated cavities. Our method mainly depends on the coexistence of transverse and longitudinal couplings between the qubit and cavity fields. Employing the longitudinal couplings, we derive a Hamiltonian which is similar to that of trapped ions interacting with two vibrational modes^33^. Using four simple interaction Hamiltonians derived from the longitudinal coupling, we design the state generation algorithm. Our algorithm can be regarded as the improved version of that in^20^ when the transverse and longitudinal couplings coexist in circuit QED systems. But it has remedied the drawback that the number of steps exponentially depends on the maximal photon number, which is replaced by a quadratic dependence. Compared with previous ones with quadratic complexity, our algorithm does not require atomic energy levels higher than two^21^,^22^, boson-number-dependent stark effects^23^,^25^,^27^, or multiboson processes of high boson numbers^22^,^24^.

When applied to the generation of NOON states, whose engineering has been extensively studied^39^,^41^,^42^,^43^,^44^,^45^,^46^,^47^,^48^, our algorithm needs only carrier and one-photon sideband transitions. Meanwhile, the number of steps only linearly depends on the maximum photon numbers. In fact, these properties for generating NOON states can be generalized to any states with a constant total photon number of both modes.

We have also discussed how to avoid the effect of unwanted terms on the generation of target state. Our numerical results show that fidelities above 0.91 can be reached in the ultrastrong regime for the two-photon evenly-populated state and NOON state when the environmental effect is neglected. The generation time can be very short, in which case, the environment has small effect on fidelities of the target states. We here note that due to the similarity of two-mode interaction Hamiltonians, the algorithm using two-mode multi-phonon processes in ref. 24 can be directly applied into our model. Thus, two-mode Fock states with high photon numbers can be generated with just two steps as one-mode Fock states in^30^.

We have noted that our method for generating NOON states is similar to a recent algorithm simplified from the one which employs Stark effects to generate arbitrary entangled states^27^.

## Additional Information

**How to cite this article**: Zhao, Y.-J. *et al*. Engineering entangled microwave photon states through multiphoton interactions between two cavity fields and a superconducting qubit. *Sci. Rep*. **6**, 23646; doi: 10.1038/srep23646 (2016).

## Supplementary Material

Supplementary Information

## Figures and Tables

**Table 1:** The fidelities 
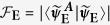
 of the target state 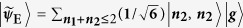 are listed for different values of the reduced driving frequency 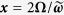 and the Lamb-Dicke parameter *η* = 2*g*_1_/*ω*_1_ = 2*g*_2_/*ω*_2_.

		*η*					
		0.2	0.3714	0.4571	0.5429	0.6286	0.7143
*x*	0.3	0.115	0.393	0.519	0.608	0.739	0.675
	0.7857	0.589	0.817	0.872	0.851	0.911	0.852
	1.0286	0.615	0.85	0.876	0.906	0.886	0.857
	1.2714	0.724	0.872	0.886	0.906	0.852	0.878
	1.7571	0.821	0.939	0.899	0.859	0.825	0.837
	2	0.867	0.915	0.838	0.876	0.887	0.859

Here 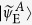 is the actually generated state using the total Hamiltonian. We have chosen the longitudinal frequency of the qubit *ω*_*z*_/2*π* = 19.5 GHz, the transverse frequency of the qubit *ω*_*x*_/2*π* = 1.2 GHz, the frequency of the first mode *ω*_1_/2*π* = 6 GHz and the frequency of the second mode *ω*_2_/2*π* = 8 GHz.

**Table 2:** The fidelities 
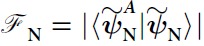
 of the target state 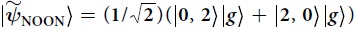 are listed for different values of the reduced driving frequency 
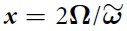
 and the Lamb-Dicke parameter *η* = 2*g*_1_/*ω*_1_ = 2*g*_2_/*ω*_2_.

		*η*					
		0.2	0.3714	0.4571	0.5429	0.6286	0.7143
*x*	0.3	0.108	0.34	0.403	0.395	0.461	0.687
	0.7857	0.675	0.815	0.833	0.862	0.829	0.868
	1.0286	0.78	0.857	0.846	0.867	0.883	0.813
	1.5143	0.871	0.877	0.873	0.877	0.787	0.806
	1.7571	0.844	0.918	0.889	0.902	0.862	0.819
	2	0.876	0.909	0.832	0.92	0.853	0.806

Here 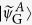 is the actually generated state using the total Hamiltonian. We have chosen the same parameters as in Table 1.

**Table 3:** Comparison of different methods for generating arbitrarily entangled states of two-mode bosonic fields.

	Pop. Leak.	No. At. Lev.	St. Eff.	Mult. Proc.
Ref. 20	Yes	2	No	L. Pn. No.
Ref. 21	No	3	No	L. Pn. No.
Ref. 22	No	3	No	H. Pn. No.
Ref. 23	No	2	Yes	None
Ref. 24	No	2	No	H. Pn. No.
Refs 25, 26, 27	No	2	Yes	None
Our proposal	No	2	No	L. Pt. No.

We use Pop. Leak., St. Eff. and Mult. Proc. to denote population leakage, the Stark effect, multiboson processes, respectively. No. At. Lev. is used to denote the number of atomic energy levels. For example, 2 denotes two energy levels when the state is generated. We use “Yes” or “No” to denote whether the population leakage (Stark effect) occurs (are used) or not. Meanwhile, L (or H). Pn. No. means multiphonon processes of low (or high) phonon number, however, L (or H). Pt. No. means multiphoton processes of low (or high) photon number.
