# Supplementary material for: Engineering entangled microwave photon states through multiphoton interactions between two cavity fields and a superconducting qubit
Source: Sci Rep. 2016 Apr 1;6:23646. doi: 10.1038/srep23646 (PMC4817062; doi:10.1038/srep23646)
Supplement: Supplementary Information [file srep23646-s1.pdf]

Supplementary information for “Engineering entangled microwave photon states through multiphoton interactions between two cavity fields and a superconducting qubit”

Yan-Jun Zhao[1], Chang-Qing Wang[1], Xiaobo Zhu[2], and Yu-xi Liu[1,3,*]

[1]Institute of Microelectronics, Tsinghua University, Beijing, 100084, China
[2]Institute of Physics, Chinese Academy of Sciences, Beijing, 100190, China
[3]Tsinghua National Laboratory for Information Science and Technology (TNList), Beijing 100084, China
[*]yuxiliu@mail.tsinghua.edu.cn

ABSTRACT

This document provides supplementary information on the approach that leads to the results presented in the main text.

1 Universal algorithm for arbitrary maximum photon numbers

The basic principle of our algorithm is state space reduction. The state space of the target state in Eq. (33) in the main text can be denoted as

$$\mathscr{H}_{2N_{\max}} = \{|n_1,n_2\rangle|g\rangle\,|n_1+n_2 \le N_{\max}\}. \tag{1}$$

We will implement $2N_{\max}$ procedures, with each procedure containing some steps.

In the 1st procedure, we aim to clear the populations in the subspace

$$\mathscr{H}'_{2N_{\max}} = \{|n_1,n_2\rangle|g\rangle\,|n_1+n_2 = N_{\max}\} \tag{2}$$

of $\mathscr{H}_{2N_{\max}}$. This can be achieved via alternatively switching $N_{\max}$ “$\bar{1}0$” transitions and $N_{\max}$ “$0\bar{1}$” transitions, i.e.,

$$\begin{aligned}&|0,N_{\max}\rangle|g\rangle \xrightarrow[f]{0\bar{1}} |0,N_{\max}-1\rangle|e\rangle \xrightarrow[f-1]{\bar{1}0} |1,N_{\max}-1\rangle|g\rangle\cdots\\ &\xrightarrow[f-2N_{\max}+2]{0\bar{1}} |N_{\max}-1,0\rangle|e\rangle \xleftarrow[f-2N_{\max}+1]{\bar{1}0} |N_{\max},0\rangle|g\rangle.\end{aligned} \tag{3}$$

to transfer the populations in the subspace $\mathscr{H}'_{2N_{\max}}$ of $\mathscr{H}_{2N_{\max}}$ to the state $|N_{\max}-1,0\rangle|e\rangle$. Thus, $\mathscr{H}_{2N_{\max}}$ is reduced to the state space $\mathscr{H}_{2N_{\max}-1}$ where

$$\begin{aligned}\mathscr{H}_{2N_{\max}-1} =&\{|n_1,n_2\rangle|g\rangle\,|n_1+n_2 \le N_{\max}-1\}\\ &\cup\{|n_1,n_2\rangle|e\rangle\,|n_1+n_2 \le N_{\max}-2\}\\ &\cup\{|N_{\max}-1,0\rangle|e\rangle\}.\end{aligned} \tag{4}$$

⋮

In the 2μth procedure, we aim to clear the populations in the subspace $\{|N_{\max}-\mu,0\rangle|e\rangle\}$ of $\mathscr{H}_{2N_{\max}-2\mu+1}$. This can be

achieved via alternatively switching $N_{\max}-\mu$ "$1\bar{1}$" transitions and $N_{\max}-\mu$ "00" transitions, i.e.,

$$\begin{aligned}
&|0,N_{\max}-\mu\rangle\,|g\rangle \xrightarrow[f-N_{2\mu}]{1\bar{1}} |1,N_{\max}-\mu-1\rangle\,|e\rangle \\
&\xrightarrow[f-N_{2\mu}-1]{00} |1,N_{\max}-\mu-1\rangle\,|g\rangle \cdots \\
&\xrightarrow[f-N_{2\mu}-2(N_{\max}-\mu)+2]{1\bar{1}} |N_{\max}-\mu,0\rangle\,|e\rangle \\
&\xrightarrow[f-N_{2\mu}-2(N_{\max}-\mu)+1]{00} |N_{\max}-\mu,0\rangle\,|g\rangle\,.
\end{aligned} \tag{5}$$

with $N_{2\mu}=2N_{\max}+(4N_{\max}-2\mu-1)\,(\mu-1)$, to transfer the populations in the subspace

$$\begin{aligned}
\mathscr{H}'_{2N_{\max}-2\mu+1} =&\{|n_1,n_2\rangle\,|g\rangle\,|n_1+n_2=N_{\max}-\mu, n_2\neq 0\} \\
&\cup\{|N_{\max}-\mu,0\rangle\,|e\rangle\}
\end{aligned} \tag{6}$$

of $\mathscr{H}_{2N_{\max}-2\mu+1}$ to the state $|N_{\max}-\mu,0\rangle\,|g\rangle$. Here Thus, $\mathscr{H}_{2N_{\max}-2\mu+1}$ is reduced to the state space $\mathscr{H}_{2N_{\max}-2\mu}$ where

$$\begin{aligned}
\mathscr{H}_{2N_{\max}-2\mu} =&\{|n_1,n_2\rangle\,|g\rangle\,|n_1+n_2\leq N_{\max}-\mu-1\} \\
&\cup\{|n_1,n_2\rangle\,|e\rangle\,|n_1+n_2\leq N_{\max}-\mu-1\} \\
&\cup\{|N_{\max}-\mu,0\rangle\,|g\rangle\}\,.
\end{aligned} \tag{7}$$

In the $(2\mu+1)$th procedure, we aim to clear the populations in the subspace $\{|N_{\max}-\mu,0\rangle\,|g\rangle\}$ of $\mathscr{H}_{2N_{\max}-2\mu}$. This can be achieved via alternatively switching $N_{\max}-\mu$ "$\bar{1}0$" transitions and $N_{\max}-\mu-1$ "$0\bar{1}$" transitions, i.e.,

$$\begin{aligned}
&|0,N_{\max}-\mu-1\rangle\,|e\rangle \xrightarrow[f-N_{2\mu+1}]{\bar{1}0} |1,N_{\max}-\mu-1\rangle\,|g\rangle \\
&\xrightarrow[f-N_{2\mu+1}-1]{0\bar{1}} |1,N_{\max}-\mu-2\rangle\,|e\rangle \cdots \\
&\xrightarrow[f-N_{2\mu+1}-2(N_{\max}-\mu)+3]{\bar{1}0} |N_{\max}-\mu-1,0\rangle\,|e\rangle \\
&\xleftarrow[f-N_{2\mu+1}-2(N_{\max}-\mu)+2]{0\bar{1}} |N_{\max}-\mu,0\rangle\,|g\rangle\,.
\end{aligned} \tag{8}$$

with $N_{2\mu+1}=N_{2\mu}+2(N_{\max}-\mu)$, to transfer the populations in the subspace

$$\begin{aligned}
\mathscr{H}'_{2N_{\max}-2\mu} =&\{|n_1,n_2\rangle\,|e\rangle\,|n_1+n_2=N_{\max}-\mu-1, n_2\neq 0\} \\
&\cup\{|N_{\max}-\mu,0\rangle\,|g\rangle\}
\end{aligned} \tag{9}$$

of $\mathscr{H}_{2N_{\max}-2\mu}$ to the state $|N_{\max}-\mu-1,0\rangle\,|e\rangle$. Thus, $\mathscr{H}_{2N_{\max}-2\mu}$ is reduced to the state space $\mathscr{H}_{2N_{\max}-2\mu-1}$ where

$$\begin{aligned}
\mathscr{H}_{2N_{\max}-2\mu-1} =&\{|n_1,n_2\rangle\,|g\rangle\,|n_1+n_2\leq N_{\max}-\mu-1\} \\
&\cup\{|n_1,n_2\rangle\,|e\rangle\,|n_1+n_2\leq N_{\max}-\mu-2\} \\
&\cup\{|N_{\max}-\mu-1,0\rangle\,|e\rangle\}\,.
\end{aligned} \tag{10}$$

$$\vdots$$

In the $(2N_{\max})$th procedure, we aim to clear the populations in the subspace

$$\mathscr{H}'_1=\{|0,0\rangle\,|e\rangle\} \tag{11}$$

of $\mathscr{H}_1$. This can be achieved via switching one "00" transitions, i.e.,

$$|0,0\rangle\,|e\rangle \xrightarrow[1]{00} |0,0\rangle\,|g\rangle\,. \tag{12}$$

to transfer the populations in the subspace $\mathscr{H}_1'$ of $\mathscr{H}_1$ to the state $|0,0\rangle\,|g\rangle$. Thus, $\mathscr{H}_1$ is reduced to the state space $\mathscr{H}_0$ where

$$\mathscr{H}_0 = \{|0,0\rangle\,|g\rangle\}\,, \tag{13}$$

is namely the intial state space for $|\psi_0\rangle$ in Eq. (34) in the main text.

Based on the above discussion, we now calculate the number of steps to generate the target state in Eq. (33) in the main text with an arbitrary maximum photon number $N_{\max}$. For alternatively switching "$1\bar{1}$" and "00" transitions, we need $f_{1\bar{1},00}$ steps, given by

$$f_{1\bar{1},00} = 1 + \sum_{N=1}^{N_{\max}-1} 2N = N_{\max}^2 - N_{\max} + 1. \tag{14}$$

For alternatively switching "$\bar{1}0$" and "$0\bar{1}$" transitions, we need $f_{\bar{1}0,0\bar{1}}$ steps, given by

$$f_{\bar{1}0,0\bar{1}} = 1 + \sum_{N=1}^{N_{\max}} (2N-1) = N_{\max}^2 + 1. \tag{15}$$

Therefore, the total step number f is

$$f = f_{1\bar{1},00} + f_{\bar{1}0,0\bar{1}} = 2N_{\max}^2 - N_{\max} + 2. \tag{16}$$

for generating the target state in Eq. (33) in the main text.
